# Removal of a Tumor Involving the Parotid and Submaxillary Glands

**Published:** 1862-06

**Authors:** E. S. Cooper

**Affiliations:** Professor of Anatomy and Surgery in the Medical Department of the University of the Pacific, San Francisco


REMOVAL OF A TUMOR *involving the Parotid and Submaxillary Glands; destruction of the Temporo-Maxillary Articulation; Reproduction of Joint, and Cure of Patient.*—By E. S. COOPER, A. M., M. D., *Professor of Anatomy and Surgery in the Medical Department of the University of the Pacific, San Francisco.*—Some of the more prominent features of the following case were given in the San Francisco Medical Press during the progress of cure. I now give it in detail.

*Case.*—Miss S. F., æt. 14, was admitted into the Pacific Clinical Infirmary, in consequence of an enlargement of two years' standing, involving the parotid and sub-maxillary glands. The tumor had grown internally until it almost filled the fauces, giving rise to great difficulty in both deglutition and respiration, and these difficulties were rapidly increasing. The tumor was nodular, and very hard on the outer side. The ramus and angle of the inferior maxilla were pressed out of their places over an inch. The face was prodigiously deformed, the length of the tumor from the outer to the inner side being over three inches.

*Operation, April* 20, 1861.—The operation was commenced by ligating the common carotid artery above the omo-hyoides muscle. An incision was then made commencing at the upper part of the one made for ligating the artery, passing directly in front and terminating one inch above the ear. A large long flap was then dissected forwards, exposing the exterior of the tumor, which was soon found to involve not only the parotid and submaxillary glands, but all intervening tissues.

The tumor being now found so much enlarged on the inner side of the jaw that it was impossible to remove it without either cutting away the ramus of the jaw or otherwise detaching the masseter, temporalis, and pterygoid muscles, disarticulating the temporo-maxillary joint, and drawing the jaw forwards to make room for extracting the tumor, the latter method was adopted. The masseter was first cut away from its attachment to the zygomatic arch with the scalpel, after which a chisel was used, and the ligaments concerned in the temporo-maxillary articulation divided. The attachments of the other muscles were also removed with the chisel, after which that side of the jaw, being set free, could readily be moved forwards to a considerable extent. The detaching of the muscles from the bone also destroyed the adhesions of the tumor to it, which were firm on the inner surface of the back portion of the ramus. This part of the bone had been absorbed by the pressure of the tumor until it was as thin as paper.

The manner of removing these attachments with the chisel may require explaining, since the instrument is seldom or never used by others for this purpose, but it is a most excellent means of detaching the soft parts from the bones in any part whatever. It is used as follows: The handle being held steadily in the hand, the edge is pressed close to the bone and moved in different directions, being constantly upon the watch that the instrument is kept between the bone and the soft parts containing any important tissue. To be more explicit; there is always a little space between the bone and important blood-vessels and nerves in the different regions of the body, and by keeping a cutting instrument directly in contact with the bone, these can be avoided. Then, as the chisel is the proper cutting instrument for the case, its edge should be carried close to the bone with a sort of gliding motion.

The soft parts being cut away from the external surface of the tumor, it was pried out with the chisel, the attachments of the parotid gland to the deep-seated parts being exceedingly fragile and easily overcome. This difficulty, however different from what usually occurs in removing the parotid gland, was the easiest part of the operation.

Having now the posterior part of the tumor detached, and the side of the jaw movable forwards to a considerable extent, I could introduce my finger sufficiently under the angle and ramus of the jaw to seize the major portion of the tumor, and draw it outwards and backwards. This being done somewhat forcibly, its attachments to surrounding parts were shown and divided until the tumor was removed. The whole operation required three-quarters of an hour.

The tumor weighed seven ounces and two drachms. It was of a fibro-cartilaginous character, and had a calculus in the center. Whether this was formed first in the parotid gland, and was the cause of the tumor or the product of it, I am unable to say. The diseased mass was completely encysted, except where it was adherent to the ramus of the jaw. This condition of the tumor aided greatly in its thorough removal.

Fortunately, the mucous lining on the inner surface of the tumor was not broken, and the hemorrhage, which was slight, from the recurrent circulation, was all discharged through the external wound.

The patient rested well during the following night, and on the next day called for nourishment, which in liquid form was swallowed without inconvenience, and the little sufferer found herself in every way comfortable.

The after treatment consisted in the application of a piece of lint over the wound, which was closed by five sutures at equal distances from each other, and a roller around the head and over the chin, as is applied in fractures of the lower jaw. This whole dressing was kept wet in an evaporating lotion, composed of one part of alcohol and ten of water. The evaporating lotion was applied every half hour, and continued for seven days, when it was discontinued, and poultices applied instead.

The major part of the wound made for ligating the carotid healed by first intention. The stitches in the upper part of the wound sloughed out in about ten days, so that the external surface of the ramus of the jaw was again in view, also the temporo-maxillary articulation, and at the end of two weeks the major portion of the exposed bone became covered with healthy granulations, so that the margins of the wound were approximated in order to promote the more rapid closure of the wound, which had rather been prevented previously until the condition of the bone was found favorable. During the operation, in breaking up the attachments of the muscles to the jaw, the periosteum was necessarily removed in several places, so that it was necessary to keep the bone in view until all was found to be right with it. In destroying the temporo-maxillary articulation, the articulating face of the condyloid process was injured, and I was unwilling to let the soft parts close over the joint until everything was healthy in or about it; and having long since discarded the idea that air admitted into joints is a source of irritation, or even of the least injury, I saw no objection to having the joint exposed to any reasonable extent.

Matters progressed very kindly, and at the end of seventeen days the commencement of the re-formation of the external lateral ligament could be distinctly noticed. In two days more, one could notice a fibrinous deposit connecting the condyloid process to the margin of the glenoid cavity, although it was so fragile that it would constantly break when the jaw was moved somewhat briskly, but soon attained strength enough to withstand the motion.

*Sloughing of Bone.*—A portion of the posterior surface of the ramus, and all the articulating face of the condyloid process sloughed, and was discharged at the end of seven weeks after the operation.

*July 26th.*—The wound is nearly entirely cicatrised, the motion of the lower jaw being almost perfect, and the deformity of the face comparatively slight.

*Sept. 7th.*—Improving in every respect. The wound is almost entirely cicatrised, and that over the temporo-maxillary articulation entirely so. This joint is so perfectly reproduced, that no one by looking at it simply could form an idea that it was ever interfered with further than was indicated by the cicatrix over it.

*Jan. 10th,* 1862.—The patient recovered in every respect, save the deformity caused by the cicatrix, and the loss of nervous power consequent upon the division of the pes anserinus, leaving that part of the face partially paralysed. This condition was constantly improving, and the tone of the parts had been so far regained that, when the mouth was quiet, no want of symmetry in the contour could be discovered on the two sides of the face. The little girl left the Infirmary to-day in excellent health and spirits, having gained seventeen pounds in weight since the operation.—*Am. Med. Times.*

